# Xerna™ TME Panel is a machine learning-based transcriptomic biomarker designed to predict therapeutic response in multiple cancers

**DOI:** 10.3389/fonc.2023.1158345

**Published:** 2023-05-12

**Authors:** Mark Uhlik, Daniel Pointing, Seema Iyer, Luka Ausec, Miha Štajdohar, Robert Cvitkovič, Matjaž Žganec, Kerry Culm, Valerie Chamberlain Santos, Bronislaw Pytowski, Mokenge Malafa, Hong Liu, Arthur M. Krieg, Jeeyun Lee, Rafael Rosengarten, Laura Benjamin

**Affiliations:** ^1^ OncXerna Therapeutics, Inc., Waltham, MA, United States; ^2^ Genialis, Inc., Boston, MA, United States; ^3^ Department of Gastrointestinal Oncology, H. Lee Moffitt Cancer Center and Research Institute, Tampa, FL, United States; ^4^ Checkmate Pharmaceuticals, Inc., Cambridge, MA, United States; ^5^ Department of Hematology and Oncology, Samsung Medical Center, Seoul, Republic of Korea

**Keywords:** pan-tumor, immunotherapy, anti-angiogenic agent, diagnostic assay, predictive biomarker

## Abstract

**Introduction:**

Most predictive biomarkers approved for clinical use measure single analytes such as genetic alteration or protein overexpression. We developed and validated a novel biomarker with the aim of achieving broad clinical utility. The Xerna™ TME Panel is a pan-tumor, RNA expression-based classifier, designed to predict response to multiple tumor microenvironment (TME)-targeted therapies, including immunotherapies and anti-angiogenic agents.

**Methods:**

The Panel algorithm is an artificial neural network (ANN) trained with an input signature of 124 genes that was optimized across various solid tumors. From the 298-patient training data, the model learned to discriminate four TME subtypes: Angiogenic (A), Immune Active (IA), Immune Desert (ID), and Immune Suppressed (IS). The final classifier was evaluated in four independent clinical cohorts to test whether TME subtype could predict response to anti-angiogenic agents and immunotherapies across gastric, ovarian, and melanoma datasets.

**Results:**

The TME subtypes represent stromal phenotypes defined by angiogenesis and immune biological axes. The model yields clear boundaries between biomarker-positive and -negative and showed 1.6-to-7-fold enrichment of clinical benefit for multiple therapeutic hypotheses. The Panel performed better across all criteria compared to a null model for gastric and ovarian anti-angiogenic datasets. It also outperformed PD-L1 combined positive score (>1) in accuracy, specificity, and positive predictive value (PPV), and microsatellite-instability high (MSI-H) in sensitivity and negative predictive value (NPV) for the gastric immunotherapy cohort.

**Discussion:**

The TME Panel’s strong performance on diverse datasets suggests it may be amenable for use as a clinical diagnostic for varied cancer types and therapeutic modalities.

## Introduction

Over the past three decades, targeted therapies have expanded the treatment options for cancer patients and oncologists (1, 2). Nevertheless, the rate and duration of response to targeted therapy varies widely among patients, both across and within clinical indications (3–5). The potential for biomarkers to improve patient outcomes and facilitate successful clinical development is quantifiable (6, 7), encouraging efforts to identify patient subtypes most likely to respond to targeted agents and to further optimize chemotherapy regimens (8, 9). Increasingly, new biomarker strategies take advantage of the convergence of high-throughput data generation, such as next generation sequencing (NGS), with the adoption of machine learning. The resulting cutting-edge biomarkers may be better suited to predict therapeutic response given the underlying complexity of cancer biology and the heterogeneity inherent to patient populations.

Initially, most targeted therapies were designed to recognize and inhibit the function of specific cancer drivers within tumor cells when dysregulated by overexpression or genetic alteration. Subsequent approved therapies also targeted biological processes and functions carried out by non-tumor constituents of the tumor microenvironment (TME), including pathological angiogenesis and immune activity. While anti-angiogenics and immune checkpoint inhibitors (ICIs) have been approved across dozens of cancer indications, both drug classes display limited efficacy, with large proportions of patients failing to receive meaningful clinical benefit, and are associated with some serious toxicities (10–12).

Despite considerable efforts, a predictive biomarker for anti-angiogenesis therapy remains elusive (13–15). Biomarkers for ICI have fared better, with various approved tests for analytes such as programmed death ligand 1 (PD-L1) combined positive score (CPS), microsatellite instability (MSI) or mismatch-repair deficiency (dMMR), and tumor mutational burden (TMB) (16). However, these biomarkers present limitations. Immunohistochemistry (IHC) assays for PD-L1 are highly variable and inconsistent between commercial laboratories and suppliers (17). MSI-H/dMMR prevalence varies substantially across solid tumor types (18, 19). Nonetheless, some patients with microsatellite stable (MSS) disease do respond to treatment so a reliable assay to capture those subsets would be useful. Similar to MSI-H, TMB assays are used to infer the potential antigenicity of tumor cells, but do not account for the functional status of immune cells in and around the microenvironment (20, 21). Other tests in development employ genomic or transcriptomic signatures to describe the state of a cancer’s immune susceptibility, typically focusing on known immune pathway genes (22–24). However, these tests have been limited in their ability to segregate responders from non-responders (25).

An alternate strategy to expand the clinical utility of targeted therapies is to model the biological state of the TME, in which interdependent biological processes contribute to stromal and tumor cross talk. We hypothesized that a TME phenotypic framework based on the two dominant biologies of angiogenesis and immune infiltration/activation could be developed as a predictive biomarker for use in patient-selected studies involving targeted therapies. A panel consisting of a complex 124-gene signature and a machine learning algorithm was constructed, trained, validated and tested following Good Machine Learning Practice guidelines (26). The model was validated with independent real world (observational data generated during routine clinical practice) and clinical trial datasets that included patient response to anti-angiogenesis or ICI therapies. Herein we describe the evaluation of the Xerna™ TME Panel (OncXerna Therapeutics Inc., Waltham, MA) to assess its potential as a commercially viable regulated device for broad use in patient-selected clinical trials. Key criteria for functionality included i) classification of patients into subtypes that predict response based on the phenotype-mechanism-of-action (MOA) theory; ii) generalization of TME features for utility across solid cancers, rather than restricting the analysis to a single cancer type; and iii) ability to classify the TME phenotypes of newly generated patient data, in addition to historical data.

## Methods

### Training and validation dataset cohorts

The overall development of the Xerna™ TME Panel utilized gene expression data and, where available, clinical outcome data, from 2723 patient samples across nine datasets including five gastric cancer, one ovarian, one colorectal (CRC), one melanoma, and one combination of gastric/ovarian/CRC. Datasets were from the public domain, commercially sourced, or proprietary. Demographic and clinical attributes of commercial datasets are provided in **Supplementary Tables 1, 2**.

**Figure 1:**
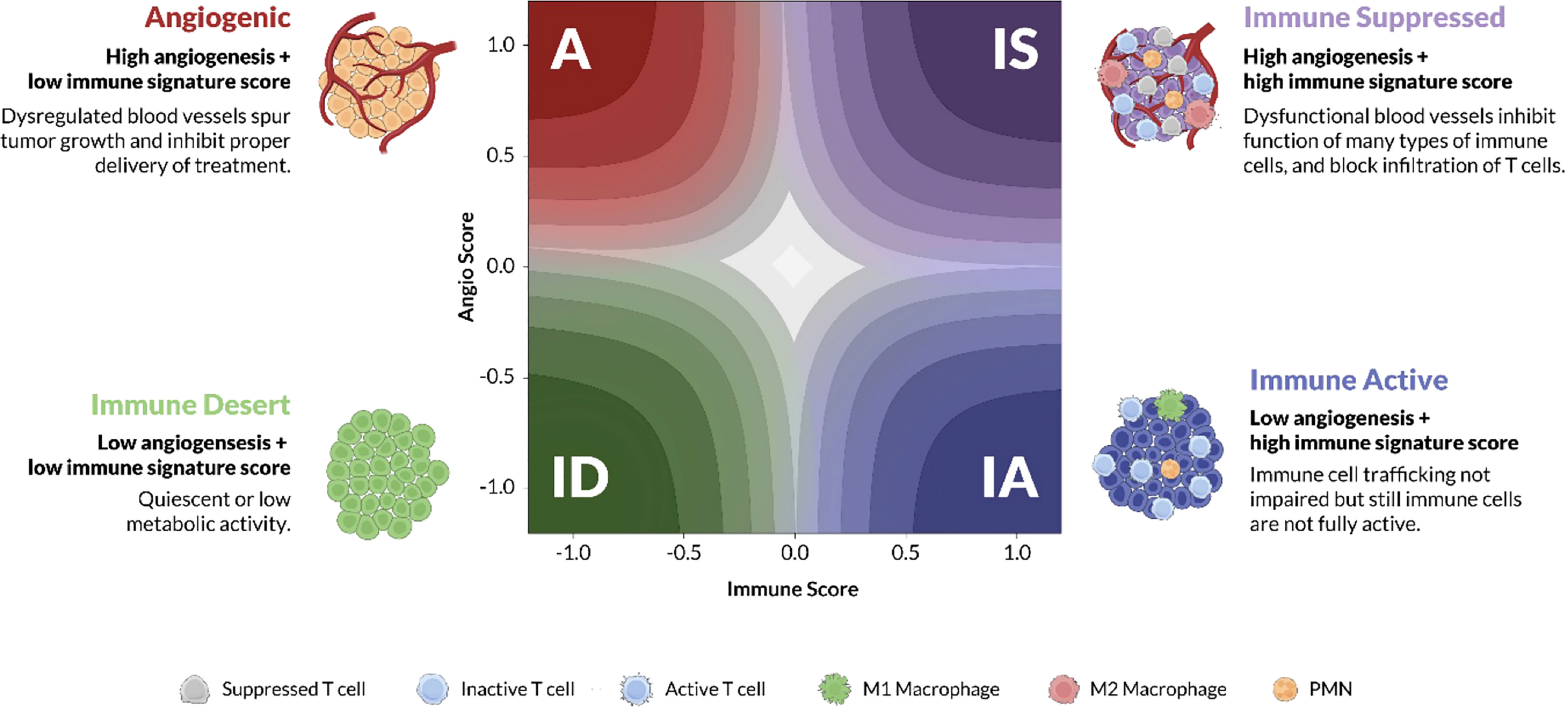
TME subtypes. Angiogenesis and immune activity are two of the dominant biological processes of the tumor microenvironment (TME). Juxtaposing signature scores for each of these biologies on a cartesian grid reveals four stromal phenotypes: Angiogenic (A), red; Immune Active (IA), blue; Immune Desert (ID), green; and Immune Suppressed (IS), purple. The x-axis represents the “immune” score while the y-axis represents the “abnormal/pathological blood vessel” score. Each phenotype is characterized by distinct molecular and pathological features, some of which are illustrated in the cartoon TMEs according to the cell-type legend. The color gradient on the latent space plot represents the probability estimate, higher probability samples are located in the darker regions towards the corners, whereas the lower probability samples are towards the center.

The training cohort consisted of 298 patients from the Asian Cancer Research Group (ACRG) obtained from Gene Expression Omnibus (GEO) (GSE62254). The ACRG dataset was selected for training as it was sufficiently large, uniformly processed on the same array platform, and included patients with a consistent treatment history with no prior targeted therapy (27). Data was combined from raw microarray expression (CEL) files and processed with expresso function from affy R package using robust multi-array average (RMA) background correction, quantile normalization, no adjustment to the PM values, and median polish summarization (28). Training labels corresponding to the four TME subtypes (**Figure 1**) were assigned based on 2 previously described complex signatures that represented pathological angiogenesis and TME Immunology. These biological subtypes were previously defined and characterized using a variety of orthogonal approaches (29). “A” subtype tumors are characterized by a dense, pathological vasculature lacking significant immune cell infiltration. “IA” tumors have an immune infiltrated tumor microenvironment marked by activated lymphocytes and M1 polarized macrophages. Conversely, the “IS” tumors have a tumor microenvironment composed of immune cell gene expression related to M2 polarized macrophages, myeloid-derived suppressor cells (MDSCs), tumor-associated macrophages (TAMs), and regulatory T cells (Tregs). IS tumors also possess a vascular phenotypes similar to the A subtype. Finally the “ID” subtype, lacks significant gene expression for either angiogenic or immune-related biologies and has a microenvironment largely devoid of dense vasculature or immune infiltration. Some of the signature genes were discovered through the study of stromal biology in a VEGF-overexpressing mouse, and therefore independent of any cancer-specific signal (29). Additional signature genes were curated by expert review of the experimental and clinical literature.

Validation datasets were procured to test whether the same model could be used to stratify patients treated with different classes of target therapy (immunogenic or antiangiogenic) in different cancer indications and (gastric, colorectal, ovarian cancer, and melanoma) and using different types of gene expression data (microarray, RNA Exome Sequencing, total RNA-Seq). Two distinct real-world gastric cancer cohorts (RNA Exome Sequencing) were obtained from Samsung Medical Center, one treated with the anti-angiogenic agent ramucirumab (Gastric-Angio), and the other treated with either pembrolizumab or nivolumab monotherapy (Gastric-Immune). A clinical trial dataset (total RNA-Seq) was provided by OncXerna Therapeutics (NCT03030287) that had been derived from an ovarian cancer cohort treated with navicixizumab and paclitaxel (Ova-Angio), and one from Checkmate Pharmaceuticals (NCT02680184) from a melanoma cohort (total RNA-Seq) treated with vidutolimod and pembrolizumab (Mela-Immune) (**Supplementary Table 1**). All four datasets included clinical outcomes defined using Response Evaluation Criteria in Solid Tumours version 1.1 (RECIST 1.1). Investigators obtained informed consent from each participant or participant’s guardian and investigations were performed after approval by a local Human Investigations Committee and in accordance with an assurance filed with and approved by the Department of Health and Human Services where appropriate.

### Feature set optimization

Prior to model training, the gene signature that would serve as model features was optimized by reducing the feature set to include only those genes that were consistently expressed across datasets representing various gene expression platforms (e.g. microarray vs. total RNA-seq vs. RNA Exome sequencing) and different tissue types (e.g. gastric vs. ovarian vs. colorectal cancers). A novel metric of “feature transferability” was developed (30) to quantify the consistency of each gene’s expression across variously sourced datasets, and to set an empirical threshold with which to remove genes from the feature set (**Supplementary Figure 1B**). The final feature set consisted of 124 genes of which a subset of genes were not weighted, roughly evenly split between the Angio and Immune subsets.

### Model training

An artificial neural network (ANN) of multilayer perceptron type with two neurons in the hidden layer was trained on the ACRG data using the final feature set and hyper parameters tuned using repeated 10-fold cross validation. The training iterates until the loss is not improving by at least 1e-4 for 10 consecutive iterations, limited to at most 1000 iterations (epochs). The resulting model consisted of three layers of nodes: an input layer, a hidden layer, and an output layer. Except for the input nodes, each node is a neuron. The neuron computes a weighted sum of its inputs, adds intercept bias, and scales the sum using a hyperbolic tangent activation function (tanh):


$$f_{i}\left(x\right)\;=\;tanh\left(w_{i}\cdot x_{i}\;+\;b_{i}\right)$$

Here $f_{i}$ is the output of the i th node (neuron), $w_{i}\cdot x_{i}$ is a weighted sum of input connections, and $b_{i}$ is the intercept bias. The hyperbolic tangent activation function (tanh) introduces non-linearity and scales the weighted sum of neuron inputs from (-∞, ∞) domain to (-1, 1) range.

Each normalized gene expression value is multiplied by weights on the connections to the two neurons in the hidden layer. Hidden neuron inputs are summed together, the intercept bias is added to the weighted sum, and the value is scaled by the hyperbolic tangent activation function (tanh). Likewise, the two outputs of the hidden layer neurons are multiplied by weights on the connections to the four output neurons. Output neuron inputs are summed together, the intercept bias is added to the weighted sum, and the value is normalized by the softmax function. The output of the model are probabilities of the four TME subtypes. The output with the highest probability estimate is chosen as the TME subtype, no minimum threshold is required to be reached. The Xerna TME model optimizes the multi-class cross-entropy loss function using the Limited Broyden-Fletcher-Goldfarb-Shanno (L-BFGS) solver.

### Model validation

Model performance was determined by classifying patients from real-world and clinical trial datasets and comparing the patient TME subtypes to clinical response. The TME Panel algorithm assigns samples to a TME subtype with an associated probability estimate. The subtype designation and its probability estimate can be used to determine the patient biomarker status. Best Objective Response (BOR) was measured in all cohorts using RECIST v1.1 criteria, and complete response (CR) or partial response (PR) were considered as responders, while stable disease (SD) or progressive disease (PD) were deemed non-responders. Prior to evaluating model performance, each dataset was normalized *via* a mapping function to the same distribution as the training data (**Supplementary Methods**). For any given dataset, certain TME subtypes were considered “biomarker positive” based on the mechanism of action of the particular therapy (defined in **Table 1** and in the **Supplementary Methods**). The model’s ability to predict clinical response based on TME subtype was reported by standard metrics: accuracy, the area under the receiver operator curve (AUROC), sensitivity (recall), specificity, positive predictive value (precision; PPV), and negative predictive value (NPV).

**Table 1:** Key performance characteristics of Xerna TME Assay across clinical cohorts.

	Biomarker/Model	ACC	AUROC	F1	Sensitivity	Specificity	Precision/PPV	NPV
**Gastric-Angio (n=48)** B+ = TME IS+A ORR(B+) =50.0% ORR(B−) = 30.8% ORR enrichment = 1.6-fold	TME	0.60 (29/48)	0.61	0.54	0.58 (11/19)	0.62 (18/29)	0.50 (11/22)	0.69 (18/26)
	Baseline	0.53	0.5	0.40	0.40	0.61	0.40	0.61
**Ova-Angio (n=32)** B+ = TME IS+A ORR(B+) = 62.0% ORR(B−) = 26.3% ORR enrichment = 2.4-fold	TME	0.69 (22/32)	0.66	0.62	0.62 (8/13)	0.74 (14/19)	0.62 (8/13)	0.74 (14/19)
	Baseline	0.51	0.5	0.39	0.39	0.59	0.39	0.59
**Gastric-Immune (n=73)** B+ = TME IS+IA ORR(B+) = 34.4% ORR(B−) = 4.9% ORR enrichment = 7.0-fold	TME	0.68 (50/73)	0.83	0.49	0.85 (11/13)	0.65 (39/60)	0.34 (11/32)	0.95 (39/41)
	IA ≥90%	0.85 (62/73)	/	/	0.54 (7/13)	0.92 (55/70)	0.58 (7/12)	0.90 (55/61)
	PD-L1=>1	0.60 (44/73)	/	0.45	0.92 (12/13)	0.53 (32/60)	0.30 (12/40)	0.97 (32/33)
	MSI-H	0.85 (62/73)	/	0.48	0.38 (5/13)	0.95 (57/60)	0.62 (5/8)	0.88 (57/65)
	Baseline	0.70	0.5	0.17	0.17	0.82	0.17	0.82
**Mela-Immune (n=38)** B+ = TME IS ORR(B+) = 53.8% ORR(B−) = 12.0% ORR enrichment = 4.5-fold	TME	0.76 (29/38)	0.75	0.61	0.70 (7/10)	0.79 (22/28)	0.54 (7/13)	0.88 (22/25)
	Baseline	0.62	0.50	0.27	0.27	0.74	0.27	0.74

Data generated from **Supplemental Table 2** for subjects that had both biomarker calls and drug response data. A simple baseline classifier served to represent the null model. The baseline classifier randomly samples the class based on prior class probabilities and simulates drug response without a biomarker. For the Immune dataset, data were available for the industry-standard biomarker PD-L1 CPS>1 as well, and its performance is given for comparison. Cells containing a “/” were not able to be calculated. Column headers represent standard machine learning performance metrics:

Accuracy (ACC), Number of correct predictions/Total number of predictions.

Area Under the Receiver Operator Curve (AUROC), Degree to which model is capable of distinguishing between classes.

Sensitivity (Recall), True biomarker responders/Total actual responders.

Specificity, True biomarker non-responders/Total actual non-responders.

Positive Predictive Value (PPV or Precision), True biomarker responders/Total predicted biomarker responders.

Negative Predictive Value (NPV), True biomarker non-responders/Total predicted biomarker non-responders.

TME, Tumor Microenvironment.

ORR, Overall Response Rate.

### Enrichment analyses

Gene set enrichment analysis (GSEA) and variation analysis (GSVA) were implemented by standard methods to examine the relationship of TME Panel subtypes to biological phenotypes (**Supplemental Materials**). GSVA, in particular, was applied to the ACRG, Singapore and TCGA STAD datasets (**Supplemental Materials**) to compare TME Panel classes to signatures of angiogenesis, inflammatory response, and immune suppression, as derived from manual curation of well-known, literature validated genes from the MSigDB Hallmark database (31).

## Results

### Xerna™ TME Panel is a complex transcriptomic model of tumor microenvironment

The Xerna TME Panel was inspired by a previously described complex signature of 3 subsets of genes representing different stages of stromal development/remodeling stimulated by an adenovirally expressed VEGF-A (29). Additional human immune-related genes were incorporated from expert evidence-based analyses, which led to a starting list that was further refined using bioinformatic tools (methods and **Supplemental Methods**). The intersection of angiogenesis and immune biologies were conceptualized as a phenotypic landscape of the TME (**Figure 1**) (32). Preliminary models using tumor sample gene expression data and z-score (population-dependent) statistical analyses sorted samples into one of the four phenotypes: angiogenic (A), immune active (IA), immune desert (ID) and immune suppressed (IS).

**Figure 2:**
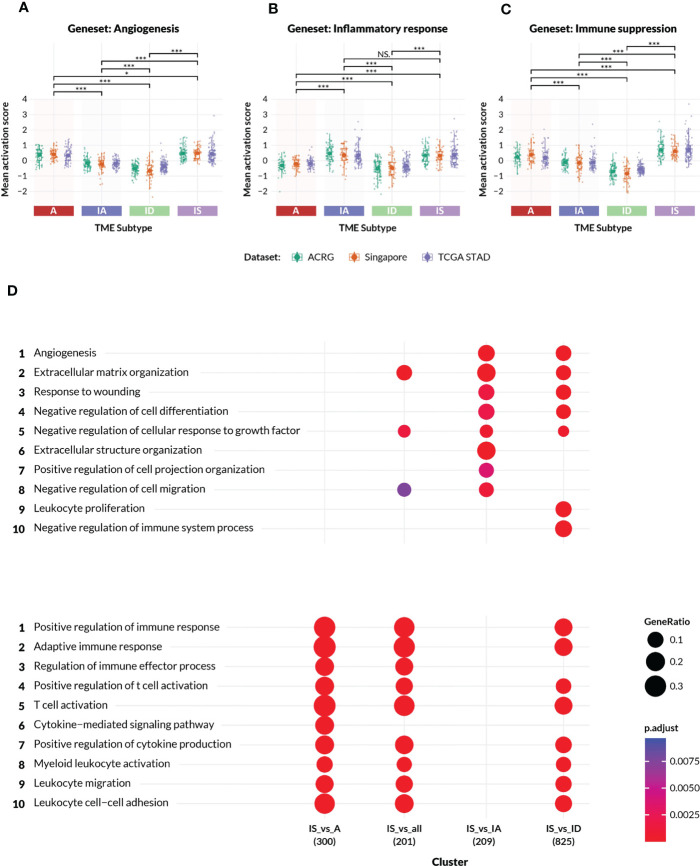
Characteristics of the TME subtypes. **(A–C)** Activation scores (y-axis) were computed on patient samples from the ACRG, Singapore Cohort, and TCGA-STAD (stomach adenocarcinoma) datasets for gene sets representing **(A)** angiogenesis and mesenchymal biology, **(B)** inflammatory response and **(C)** immune suppression, respectively. The gene sets were manually derived subsets of the GSEA MSigDB Hallmark collections, and are listed in the **Supplementary Methods**. In each plot, the datasets are colored according to the legend, and grouped by TME subtypes A, IA, ID and IS. All pairwise comparisons between TME subtypes were analyzed for statistical differences in mean activation score by a t-test, with (*) indicating p-value< 0.05, (***) indicating p-value< 0.0005, and “NS” indicating no significant difference. **(D)** Gene Ontology enrichment analysis confirmed the distinct biologies of genes differentially expressed between samples in **Supplementary Table 1** excluding Mela-Immune from the IS subtype versus each of the A, IA and ID subtypes. The number of differentially expressed genes is shown beneath the contrast label. The top ten angiogenic and immunogenic gene ontology process terms are respectively listed with an adjusted p-value of between<0.0025 and 0.0075.

Each biological phenotype was hypothesized to indicate a therapeutic strategy based on the drug’s MOA. For example, samples classified as the A or IS phenotypes which both show features of cardiovascular/endothelial cell biologies (**Figures 1**, **2**), were hypothesized to have more favorable responses to an anti-angiogenic, whereas IA or IS samples, with features of immune cell/cytokine biology would be hypothesized to respond best to immune based therapies. These were the *a priori* hypotheses that lead to different definitions of a biomarker status in different clinical contexts.

**Figure 3:**
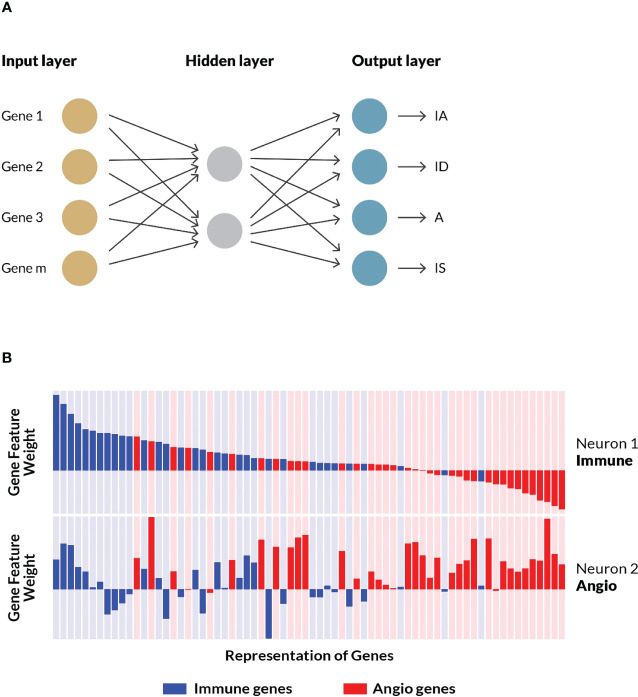
ANN model training and examination. **(A)** A shallow artificial neural network model performed the best in initial benchmarking tests against various other algorithms, specifically logistic regression and random forest. The model consisted of an input layer of genes and their (normalized) expressions, a hidden layer with two nodes, and an output layer assigning probabilities that a given sample belongs to each of the four TME subtypes (i.e. stromal phenotypes). See **Supplemental Methods** for additional details. **(B)** A schematic representation of a subset of the weights and associated biological processes of matched genes across both neurons. For each node in the hidden layer—neuron 1 and neuron 2—gene weights are represented as bars corresponding to the magnitude and direction of the values learned by the trained model. Genes curated as associated with immune processes are colored blue while those associated with angiogenesis biology are colored red. Neuron 1 was characterized by high, positive weights for immune genes, while Neuron 2 was characterized by high, positive weights for angiogenesis genes. The same gene order is displayed for both neurons, illustrating how some genes are positive on both neurons, while others flip signs. A subset of the final geneset is shown.

An *in silico* experimental design was conceived for the construction, training, testing and independent evaluation of a computational model that could predict TME subtypes (**Figure 1**) based on gene expression data from tumor samples. An artificial neural network was trained on 298 real-world gastric cancer patient samples collected by the ACRG consortium using an optimized 124 gene feature set. The final model that constitutes the TME Panel consisted of: the input layer, i.e. gene signature (features); the hidden layer, i.e. two neurons, each with weights for all genes; and the output layer, i.e. four neurons representing the four TME subtypes, and their associated probabilities (**Figure 3A**).

To better understand how the model learned to associate the input gene set with the stromal phenotypes, the weights assigned to individual genes on each of the two hidden neurons were examined (see schematic representation in **Figure 3B**). Neuron 1 preferentially gave high, positive weights to genes associated with immune processes, and low, negative weights to genes associated with angiogenesis. Conversely, neuron 2 generally gave high, positive weights to angiogenesis-associated genes, and low, negative weights to immune-related genes. These gene-biology associations were never exposed to the algorithm during training. Rather, the ANN model independently learned gene weights that corresponded to the two biological processes represented in the feature set. Further inspection of the hidden layer gene weights suggested these biologies are interconnected, with some genes showing high, positive weights, or low, negative weights on both neurons, which is consistent with our understanding of the crosstalk between immune cells and vascular cells (33) as well as their common embryonic lineage (**Figure 3B**).

### The Xerna TME Panel subtypes are defined by their expression of immune and angiogenic related genes

The TME Panel learned the complex gene signature associated with two distinct biological processes—immune and angiogenic—whose intersection classifies four subtypes. To confirm that the TME subtypes represented the inferred stromal phenotypes, enrichment analysis was performed using gene sets derived from the MSigDB Hallmarks collections. A subset of the Hallmark genes was selected to represent each of the following biologies: angiogenesis; inflammatory response; and immune suppression. Activation scores were computed for these gene sets across three independent datasets and compared between all pairwise TME subtypes (**Figures 2A–C**).

The observed activation score patterns generally agreed with the TME Panel subtype designations. The median angiogenesis signature score was positive (median range of approximately 0.3 to 0.5) in A and IS subtypes, and negative (median range of approximately -0.2 to -0.55) in IA and ID subtypes (**Figure 2A**). The median inflammatory response activation scores were (median range of approximately 0.45 to 0.2) in IA and IS subtypes compared to (median range of approximately -0.2 to 0.-0.4) in A and ID subtypes (**Figure 2B**). Furthermore, the immune suppression gene set scores were highest among the IS subtype (**Figure 2C**). The statistical significance of these patterns was inferred by pairwise t-test, and is shown in **Figures 2A–C**.

The IS subtype was of special interest since these patients express characteristics of both dominant biologies, and may be underserved by currently available targeted therapies. To better understand the biological processes that defined the IS subtype, differential gene expression analysis was performed on ACRG patient samples from the IS subtype versus those from each of the other TME classes, followed by gene ontology enrichment (**Figure 2D**). Canonical immune-related GO biologies such as “regulation of immune effector process” and “cytokine-mediated signaling pathway” were differentially enriched between IS and A subtypes, but were not distinct between IS and IA. Typical stromal-related GO biologies such as “extracellular matrix organization”, “angiogenesis”, etc., were differentially enriched between IS and IA subtypes, but were similar between IS and A. IS samples were differentially enriched for most of the GO biologies when compared to the quiescent ID subtype. Thus, patient samples classified as IS simultaneously displayed gene expression characteristics of two orthogonal but potentially interfering biologies.

**Figure 4:**
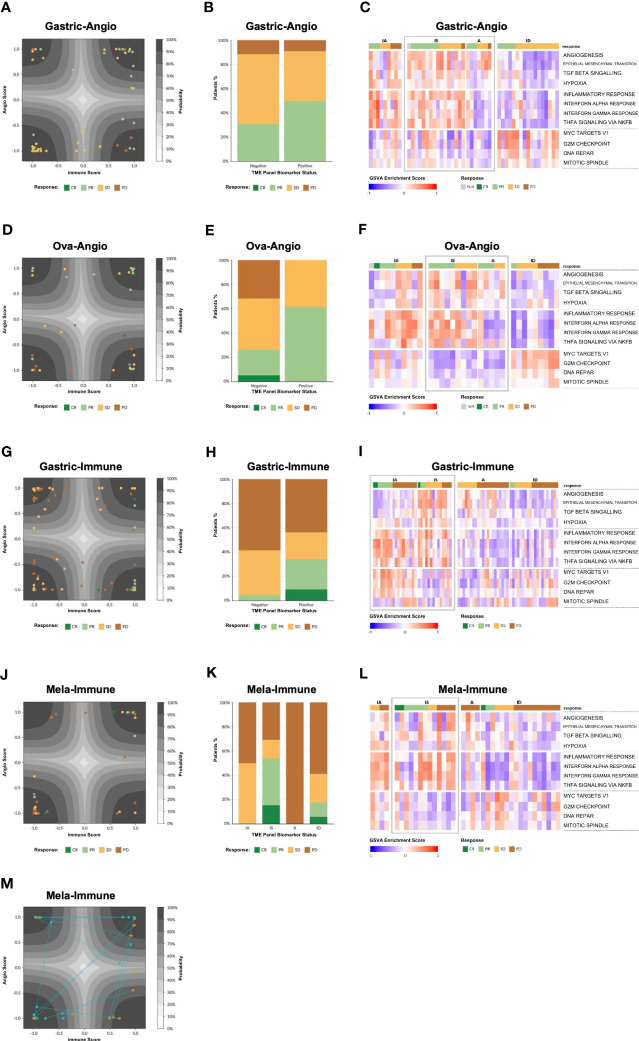
Model validation. **(A)** Patient tumor samples from the Gastric-Angio cohort projected on the latent space of the TME Panel classifier. The latent space is a two-dimensional representation of the two neurons in the hidden layer of the model, with neuron 1 as the x-axis and neuron 2 as the y-axis. The axis ticks correspond to neuron scores. The gray contours create a probability estimate gradient, as indicated in the legend. Each glyph is a patient sample, colored according to RECIST score, according to the legend. **(B)** Tumor response tabulated based on RECIST score. Biomarker status for each cohort is defined in **Table 1**. **(C)** Gene Set Variation Analysis (GSVA) showing enrichment of various pathways (rows) for each patient in the Gastric-Angio cohort (columns). Patients are grouped on the x-axis by TME subtype and tumor response based on RECIST criteria, shown according to the legend. GSVA signatures are grouped on the y-axis by general biological class with angiogenesis-related biology on top, immune-related biology in middle, and cell cycle and proliferation-related biology at the bottom. TME subtypes that correspond to the biomarker-positive status for each cohort are boxed in “grey” color. **(D–L)** Analyses of the other cohorts, as in **(A–C)**. **(M)** Analysis of matched pre and post treatment samples. Post treatment samples were taken Week 3 Day 1 of treatment. The arrows demonstrate how the sample has changed after treatment.

Model performance is reported in **Table 1**. Most responders to anti-angiogenic agents were found among high angiogenesis TME subtypes (**Figures 4B, E**), whereas most responders to ICIs were found among high immune TME subtypes (**Figure 4H**). Most responders to combination immune therapy were found in the immune suppressed group (**Figure 4K**). These observations were consistent with the hypothesis that underlying biological phenotype could predict targeted therapy outcome. Generally, individual hallmark GSVA signatures are not sufficiently predictive of drug response (**Figures 4C, F, I, L** and **Supplemental Figure 2**), unlike the collection of genes captured in the TME panel. However, these hallmark GSVA signatures do reveal a general correspondence between pathway enrichment and TME subtype, most evident in high expression of angiogenesis-related signatures in the A and IS subtypes and high expression of immune-related signatures in the IA and IS subtypes. Cell cycle and proliferation gene signatures were most evident in the ID subtype.

To visualize the distribution of patient samples among the TME subtypes and their associated probabilities, a “latent space plot” was developed. In brief, each sample was graphed using the hidden neuron 1 value on the X-axis and hidden neuron 2 value on the Y-axis (**Figures 4A, D, G, J**). Since these axes were shown to correlate with high weighting of genes associated with immune biology and angiogenesis biology, respectively, the latent space plot is analogous to the four stromal phenotypes shown in **Figure 1**, with the X-axis generally representing an immune score and the Y-axis generally representing an angiogenesis score. The distance of a sample from the center of the plot is correlated with the TME output layer probability estimate, with high probability estimate calls closer to the corners, and low probability estimate calls closer to the center. Additional patient data, including BOR, PD-L1 status, and other known factors, can be layered on the visualization to facilitate model interpretation.

### The Xerna TME model enriches for patient responses to tumor microenvironment-directed therapies in real-world and clinical trial cohorts from multiple cancer types

Correlations of TME subtypes to clinical benefit were calculated using data provided in **Supplemental Table 2**. Biomarker designations for each cohort were determined prior to analyses on the basis of the respective therapeutic mechanism-of-action.

#### Response to anti-angiogenic treatment

Gastric-Angio and Ova-Angio cohorts were analyzed to determine if TME subtype classification could predict response to anti-angiogenic therapies. Patients classified as high angiogenic subtypes (A+IS) were expected to respond to ramucirumab or the anti-VEGF/anti-DLL4 bispecific navicixizumab plus paclitaxel, respectively. Thus, A+IS patients were designated as biomarker-positive (B+). Of the 32 patients with response data in the Ova-Angio, 13 were scored as B+ and the overall response rate (ORR; ORR = CR+PR) was 62%, a 2.4x improvement over the B− group (**Table 1**). TME panel analysis yielded an Area Under the Receiver Operator Curve (AUROC) of 0.66 with sensitivity of 0.62, specificity of 0.74 and NPV of 0.74. Disease control rate (CR+PR+SD) was 100% among B+ patients. The Gastric-Angio dataset included 22 B+ and 26 B− patients, with an ORR of 39.6%, a B+ response rate of 50.0% and a B− response rate of 30.8%. The TME model yielded an AUROC of 0.61, with sensitivity of 0.58 and specificity of 0.62. In these and all other assessment metrics, the TME Panel performed superior to the null model in differentiating responders from non-responders. A baseline response to paclitaxel is expected in both cohorts, suggesting that enrichment of responders to the anti-angiogenic agents in the B+ group may be under-represented. Together these results indicate that patient stratification with this biomarker facilitates the identification of patients responsive to anti-angiogenic therapy.

#### Response to immune checkpoint inhibition (ICI)

In the Gastric-Immune cohort, patients with high immune axis subtypes (IA+IS) were hypothesized to be more responsive to treatment than those with low immune axis subtypes (A+ID). Thus, in this case, IA+IS patients were designated as B+. Thirty-two of 73 patients were characterized as B+, and 41 of 73 were B−. The ORR was 20.6%, with response observed in 34.4% of B+ patients, and 4.9% among B− patients. The TME Panel predicted response to ICI therapy with an AUROC of 0.83, with a sensitivity (recall) of 0.85 and specificity of 0.65. The NPV, i.e., the ability of the model to discriminate non-responders, was 0.95 (**Table 1**). Overall, the TME Panel results were similar to those of the canonical ICI biomarker PD-L1 CPS ≥ 1, results for which were available in the patient sample metadata. When using MSS to further stratify patients, the ORR was 15.4%, with 32.1% in biomarker-positive, and 2.7% in biomarker-negative. This observation is noteworthy since historical rates of response in MSS are low for ICIs; such patients are often not eligible for ICI therapy. As expected, a high ORR was noted in MSI (5/8 or 62.5%), with 100% ORR of biomarker-positive MSI compared to 25% in biomarker-negative MSI (**Table 1**). Performance characteristics could be further tuned by setting additional thresholds based on TME subtype probability estimate. For example, defining B+ only as a high probability estimate (≥90%) IA subtype resulted in an ORR of 58% (7 responders out of 12 patients in this subset), with sensitivity of 0.54 and specificity of 0.92 (**Table 1**). While using this single subtype and threshold for biomarker positivity provides the highest accuracy and precision for the TME panel, there is an overall lower sensitivity. Therefore, depending on the desire to maximize specificity, sensitivity, and/or precision, the employment of different thresholds may provide a flexibility that should be considered for future clinical development as a biomarker.

#### Response to combination immunotherapy treatment

Patients participating in the Mela-Immune cohort were refractory to ICI and given vidutolimod plus pembrolizumab. With this treatment regimen, the IS subtype was hypothesized to be associated with response and therefore identified as B+. Thirteen of 38 patients were B+, and for them ORR was 53.8% (compared to the overall ORR of 26.3%). The TME Panel yielded an AUROC of 0.75, sensitivity of 0.70, specificity of 0.79, and NPV of 0.88 (**Table 1**). Seven of the 10 partial or complete responders were classified in the IS (B+) subtype, while the other three were observed in the ID subtype. This distribution of responses among the IS and ID phenotypes is consistent with the hypothesized MOA of vidutolimod in activating an immune response through the TLR9 pathway. This cohort also allowed for the analysis of treatment effects in 15 patients with paired pre- and post-treatment samples. Of these, 5 patients transitioned from ID to the more immunogenic states IA (n = 3) and IS (n = 2) (**Figure 4M**).

### Xerna™ TME panel yields a clear decision boundary between B+ and B−

**Figure 5:**
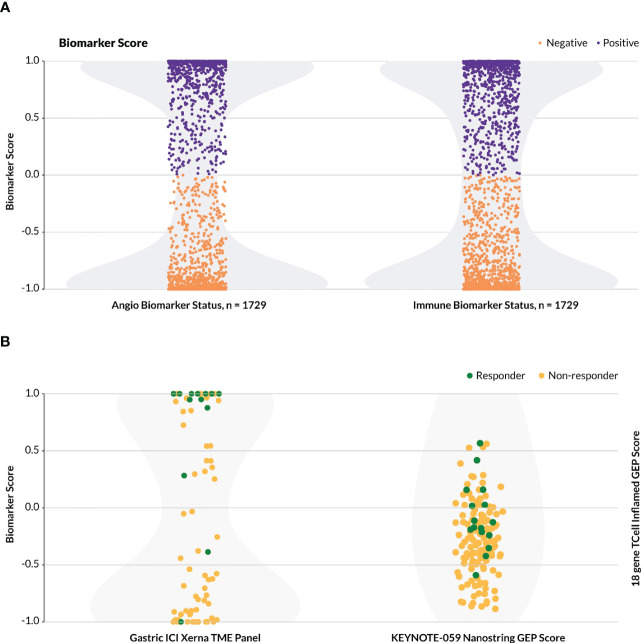
Distribution of biomarker calls. For the Xerna TME Panel, biomarker score is a cumulative probability estimate of the TME subtypes considered biomarker positive. **(A)** The distribution of TME Panel biomarker calls for both an immunotherapy (IA+IS = positive, A+ID = negative) and anti-angiogenic (A+IS = positive, IA+ID = negative) for all datasets in **Table 1**, excluding Mela-Immune. Shading in light gray indicates sample density. **(B)**Biomarker scores for two datasets. Shading in light gray indicates sample density. (Left) Biomarker scores for the Gastric-Immune dataset follow a bimodal distribution. Samples with y ≥ 0 are designated as B+, and the rest are B-. (Right) The KEYNOTE/activation-score based model, including 144 patients with gastric cancer, does not follow a bimodal distribution, and may require a zone of no confidence in which patients cannot be scored as biomarker positive or negative.

The Xerna TME Panel was trained on features that are generally involved in stromal and immune biologies, irrespective of the tissue of origin of a tumor. Thus, the model was hypothesized to be capable of classifying patient samples from any solid tumor into a TME subtype that potentially can help to identify an appropriate therapy. To date, nearly 5,500 samples from various datasets have been analyzed by the TME panel, ranging from gastric, ovarian, colorectal, melanoma, breast, liver to prostate cancer datasets. The majority of patients (~80%) across the cancer types and datasets were assigned to one of the four TME subtypes with high probability estimate (≥ 0.8). In the event the biomarker status is a sum of two TME subtypes, e.g. IA+IS, the combined B+ probability estimate is higher still than the single subtype probability estimate. By its nature, the TME Panel yields a bimodal distribution of biomarker positive versus negative calls for any particular phenotype-MOA hypothesis with a clearly delineated decision boundary (**Figure 5A**).

In the Gastric-Immune dataset, for example, B+ was defined as a combined probability estimate of IA+IS > 50%, and this threshold captured all but two true responders (**Figure 5B**, left). This stands in contrast to a typical activation-score based model, or a (near-)normal distribution of biomarker probabilities, in which the greatest frequency of patients score in the middle, and which may include a zone of no (or low) probability estimate in assigning biomarker status (9). In one such example (**Figure 5B**, right), the T cell inflamed 18-gene expression profile (GEP) was used to assess patients that received pembrolizumab in various KEYNOTE trials (22). In the KEYNOTE-059 gastric cancer study, which evaluated the anti-tumor activity of pembrolizumab monotherapy in a similar patient group as the Gastric-Immune RWD cohort, the authors described the GEP as having a higher score in aggregate for responders compared to non-responders, but did not define a discrete threshold for biomarker-positive or -negative patients (25).

### Xerna™ TME panel is prognostic in a CRC cohort

One challenge in assessing the predictive power of the TME Panel, or any biomarker, is access to datasets with relevant treatment and patient outcomes. However, one may gain a sense of the model’s potential by examining its prognostic ability in clinical settings in which certain disease phenotypes are known to be associated with better/worse outcomes. For example, previous work in CRC has shown that patients presenting with pathologically angiogenic tumors have less favorable outcomes while immune responsive tumors derive the most clinical benefit (34–37).

To test whether TME subtypes were prognostic of survival in CRC, gene expression data annotated with survival was analyzed from the CIT CRC dataset (Supplementary Methods, **Supplementary Figures 3A, B**). In both early (0–2) and late (3-4) stage CRC, the A subtype had the shortest recurrence free and overall survival, respectively, while the IA subtype had the longest RFS and OS. The IS subtype also showed less favorable clinical prognosis, consistent with the poor outcomes associated with pathological angiogenesis. Thus for this dataset, the TME Panel was prognostic for survival.

## Discussion

Whether a TME-targeted cancer therapy yields a clinically beneficial response depends on the biological context of each patient’s TME. We set out to develop a biomarker panel that could predict response to various approved and investigational TME-targeted drugs, irrespective of the specific tumor type. To accomplish this, we identified dominant biological processes that define stromal phenotypes, established a complex gene signature that could be measured robustly by RNA-sequencing, and trained and validated a machine learning model to classify patients into one of four TME subtypes. By analyzing multiple independent datasets from real-world and clinical trial settings, we showed that the TME subtypes could enrich for response and discriminate against non-response based on the MOA of a particular therapy. The model demonstrated the ability to enrich for therapy-responsive patient subgroups in gastric cancer, ovarian cancer and melanoma, for both angiogenesis inhibitors and immune modulators. Moreover, it has potential as a prognostic marker as demonstrated with an example in colorectal cancer, similar prognostic potential has been observed in other tumor types (38), however, further validation is required. Ongoing work will determine if the Xerna™ TME Panel can also be used to predict therapeutic response in CRC and numerous other indications.

The first key innovation of the TME panel was implementing an approach to biomarker modeling that facilitated hypothesis testing and interpretation (**Figure 1**). The idea was to model multiple biological processes to classify patients into phenotypic subgroups, where each subgroup then has an associated therapeutic hypothesis, rather than attempt to derive a signature retrospectively from drug responses. Transcriptomics data was chosen as the most appropriate modality to classify TME phenotypes due to the high throughput of this data type, as well as the ability to generate consistent data with widely available technologies. Several academic and for-profit companies have successfully transitioned RNA-based gene expression signatures into prognostic tools (39), such as the consensus molecular subtype for characterization of colorectal cancer (40) and MammaPrint (41) and Oncotype DX (42) assays for breast cancer recurrence. Recently, another TME-focused complex transcriptomic biomarker was described (43). Deriving a host of immune and fibrotic signatures, it classified patients into four distinct phenotypes that correlated with immunotherapy response in melanoma, bladder, and gastric cancers. The investigators concluded that the complexity and redundancy of features was important for robust performance of the model, because no one signature alone was consistently correlated with response in all tested cohorts. **Supplementary Figure 2** demonstrates the predictive performance of GSVA genesets across the four validation cohorts. In the majority of analyses the Xerna TME panel has superior predictive performance. In the case of the Angiogenesis geneset predictive performance in the Ova-Angio cohort is comparable to the Xerna TME panel, however, this same geneset does not yield meaningful results when applied to the Gastric-Angio cohort. The Xerna TME panel generally has superior performance to the GSVA genesets and has a broader applicability to more diverse tissue types (**Supplementary Figure 2**). This study further shows that biomarkers representing complex biology may prove broadly applicable for predicting response to cancer drugs.

The chosen model architecture, a shallow artificial neural net with a two-neuron hidden layer, was chosen for its relative simplicity to mitigate overfitting risk and enable interpretation (in contrast, for example, to a deep neural network or graph-based architecture), while still sufficient to learn non-linearities and the interconnected relationship between angiogenesis and immune biologies that was not hypothesized *a priori*. Unlike many other RNA signature scoring methods that are computed on the distribution of input data (“population-based”), such as a z-score (44), the TME Panel can analyze new data inputs without adjusting the training data distribution. Therefore it may be used in regulated testing environments such as clinical devices in which the trained algorithm must remain unchanged. The outputs are robust to various technical and biological sources of variance, and binary, enabling clear decision boundaries. Furthermore, a flexible biomarker logic can be employed with the TME Panel *via* the assignment of a single subtypes or combination of subtypes to underlie therapeutic hypotheses. We propose that the use of a machine learning approach, albeit a relatively simple one, is essential to the model’s ability to (a) accurately classify biologically relevant TME subtypes, with (b) high confidence decision boundaries, for (c) retrospective and prospective data without having to re-compute the underlying data distribution or retrain the model. Each of these aspects of the TME Panel provides significant advantages in its continued development as a precision medicine platform.

Not only does the TME Panel classify patients into biologically defined subgroups, but those phenotypes are also therapeutically relevant for multiple diseases and classes of therapy. The broad applicability of the Xerna TME panel to a wide range of tumor types is achieved through several of its design elements. Firstly, as the inspiration for the initial gene expression sets involved an adenoviral-driven VEGF construct to drive a pathological microenvironment in the absence of any tumor cells, this assured that no tumor-specific features would dominate gene selection. This also allowed for the establishment of biological classes defined solely by the cells of the microenvironment, rather than by tumor cells, which frequently dominate and confound gene signatures with their expression profiles. The four biological subtypes defined by this panel are common to all solid tumors, albeit likely with differing prevalence across tumor types. Finally, the careful assessment and curation of genes using feature transferability allowed genes to be removed on the basis of having too low, high or variable of an expression across a variety of tumor types, including colorectal, gastric, and ovarian cancers.

TME classification yielded 1.6 to 7-fold enrichment (**Table 1**) of clinical benefit for the indications evaluated in this study. This includes the anti-angiogenic navicixizumab, in combination with paclitaxel, in platinum-resistant ovarian cancer patients; monotherapy treatment with an immune checkpoint inhibitor in MSS gastric tumors; and treatment of immune checkpoint-resistant melanoma patients with the combination of the immunomodulator vidutolimod and pembrolizumab. Additionally, in a pre-planned analysis that was part of a Phase 2 gastric cancer combination study (NCT0409641 (45)) with the anti-phosphatidylserine antibody bavituximab and pembrolizumab, the TME Panel showed a 5.5-fold enrichment of response when comparing the immune-high subtypes (IA+IS) to immune-low subtypes (A+ID) (data not shown).

While these analyses demonstrate potential to expand the use of TME-targeted oncology drugs, there are still avenues to be explored with the TME Panel. For example, a more complete understanding of the immune desert “ID” phenotype may provide guidance for therapies such as tumor vaccines or other immune modulators. Gene set variation analysis of the four different cohorts identified a preponderance of G2M cell cycle, Myc-driven biology and DNA repair signatures in the ID subtype, suggesting that chemotherapies alone could provide the best overall clinical benefit to patients with this tumor subtype. Cell-cycle and proliferation signals prevalent in the ID subtype may account for the responses observed in the Gastric-Angio and Ova-Angio cohorts which included paclitaxel in the treatment combination.

Across all tumor types tested, the prevalence of each of the four TME subtypes are relatively common with 15-40% of samples represented in each subtype. This contrasts with the majority of NGS-supported companion diagnostics which identify rare DNA alterations and support a single class of therapy. The TME Panel can also be viewed as complementary to other tissue-derived DNA and protein-based biomarkers and can be combined with other platforms to refine therapeutic hypotheses. For example, a full NGS characterization of tumors may be performed to evaluate specific DNA mutations while RNA can be analyzed by the TME Panel, thereby maximizing the information content of precious patient samples. Combining the TME Panel outputs with other orthogonal methods like IHC could help identify the subset of patients for whom specific treatments are most beneficial. One such hypothetical example includes using PD-L1 status in combination with the Xerna TME immune-high subtypes (IA+IS) to predict the best responders in the Gastric-Immune cohort. While the PD-L1-positive ORR is 20.6% for the entire MSS portion of the cohort, combining it with the immune status enables an identification of all PD-L1 positive responders in the Immune-high subtypes (44% ORR) vs. no responders (0% ORR) in the PD-L1-positive Immune-low subtypes (data not shown). Using this approach, the TME Panel could help unify patient profiling for immune-targeted therapies, which currently relies on assays with disparate scoring methods (MSS/MSI, MMR, TMB, and PDL-1). Finally, the TME Panel may be used to address key therapeutic opportunities that lack predictive biomarkers entirely, e.g. anti-angiogenic agents, cancer vaccines or even chemotherapies.

A few caveats and limitations to the studies presented here should be noted. First, the clinical cohorts analyzed are relatively small in size (ranging from ~30-70 subjects) and larger cohorts will be important to continue to explore the predictive capabilities of this panel. Secondly, the performance of this biomarker panel may be suboptimal for some cohorts, especially the Gastric-Angio. In combinations of microenvironment-targeted therapies, such as ramucirumab (anti-VEGFR2 mAB) and paclitaxel, it may be more difficult to ascertain the TME-specific effects of a therapy in the midst of a chemotherapy. For example, in the RAINBOW trial in second-line and beyond gastric cancer, paclitaxel provides an approximate 16% response rate while its combination with ramucirumab provides 28% (46). Therefore, enrichment of responses in the biomarker-positive TME subtypes are likely to be diminished since the chemotherapy provides some significant clinical benefit on its own. Finally, the use of archival tissues (which were employed in the Gastric-Angio and Ova-Angio cohorts) may not be as optimal as pre-treatment biopsies (used for the Gastric-Immune and Mela-Immune cohorts) since intervening therapies may have an effect on the tumor microenvironment. A better biomarker performance may therefore be expected when the most contemporary assessment of microenvironment features may be made.

In conclusion, the Xerna™ TME Panel represents an advance in precision medicine, particularly in the support of TME-targeted therapies. Continued development and testing of this panel in retrospective and prospective clinical cohorts is underway and it has already been analytically validated as a clinical trial assay in a CAP-accredited lab. The Xerna™ TME Panel is currently being commercialized as a research use only assay by diagnostic companies (47, 48) and will be available for use in clinical trials. Additional exploration is underway to apply a similar platform approach to model additional “hallmark” biologies that can predict response to other targeted therapies.

## Data availability statement

The following datasets were obtained from Gene Expression Omnibus (GEO): GSE15459, GSE39582, and GSE62254. The data from clinical trials NCT0303028 and NCT02680184 are part of ongoing clinical development programs and are kept confidential. Further, the RWD from Samsung Medical Centre are not publicly available due to patient privacy requirements. Any further inquiries may be directed to the corresponding author.

## Ethics statement

Investigators obtained informed consent from each participant or participant’s guardian and investigations were performed after approval by a local Human Investigations Committee and in accordance with an assurance filed with and approved by the Department of Health and Human Services where appropriate. The patients/participants provided their written informed consent to participate in this study.

## Author contributions

MU, DP, SI, LA, MŠ, RR, and LB were involved in the conception of the studies, data analysis/interpretation, drafting/revising of the manuscript, and project leadership. RC and MŽ performed data analysis/interpretation and manuscript revisions. KC, VS, and BP provided key scientific input on data analysis/interpretation, quality control/review of data, and critically reviewed the manuscript. MM, HL, AK, and JL provided data and key scientific input and critically reviewed the manuscript. All authors contributed to the article and approved the submitted version.
