# Supplementary material for: Xerna™ TME Panel is a machine learning-based transcriptomic biomarker designed to predict therapeutic response in multiple cancers
Source: Front Oncol. 2023 May 12;13:1158345. doi: 10.3389/fonc.2023.1158345 (PMC10213262; doi:10.3389/fonc.2023.1158345)
Supplement: Supplementary file 1 [file DataSheet_1.pdf]

Supplementary Material

Supplementary Methods

Xerna TME Panel Overview

The Xerna TME panel includes roughly 100 gene features derived from experimental and clinical evidence that capture a broad readout of stromal biology (1–3). The model algorithm was trained to learn the dominant biologies represented by the gene features, rather than learning a specific tumor histology or attempting to fit a particular therapeutic response. Special care was taken to avoid overfitting, including the application and invention of various feature reduction methods, selection of a relatively simple algorithm architecture, and most importantly, validation on numerous independent datasets. The method coined “feature transferability” not only had the effect of pairing down the total number of gene features to minimize overfitting, but it helped limit the features to those applicable across multiple tumor types and different gene expression assay platforms, thus boosting the robustness of the panel.

Algorithm Selection

Three types of machine learning (ML) algorithms—Logistic Regression (LR), Random Forest (RF), and Artificial Neural Network (ANN)—were considered initially. All three were trained on the ACRG dataset and hyper parameters tuned with 10-fold cross validation. Training used the same 125 gene signature as previously employed in the “population-based” Z-score model (2). All gene expression values of training and validation datasets were preprocessed using quantile transformation to a uniform distribution between 0 and 1. The ML models were then compared in terms of the ability to predict clinical response to targeted therapy in the Gastric-Angio and Gastric-Immune datasets. The ANN algorithm displayed the highest sensitivity and specificity in these real-world data and thus was selected for further development.

Enrichment Analyses

Gene set enrichment analysis (GSEA) aggregates the per gene statistics across genes within the gene set and enables detection of small but coordinated changes. The goal of GSEA is to determine if genes from a predefined set are randomly distributed throughout the ranked gene list or primarily found at the top or bottom of the list. The GSEA implementation in the cluster Profiler R package, v3.18.1 was used. For differentially expressed genes ($FDR < 0.05$, $|LFC| > 1$) gene lists were sorted based on log2 fold change values. The cutoff for adjusted P-values

(Benjamini-Hochberg procedure) was set at 0.05. The results of the analysis were visualized using the plots provided in the package.

The combined z score method (4), which considers z scores together for each signature set at each individual sample, was used to compute an activation index. Although there are several published measures for activation of pathway or signature in a given tissue(5), the combined z score method was selected because it is intuitive and may be platform independent. The z score represents the difference in standard deviations between the error-weighted mean of the expression values of the genes in a pathway and the error-weighted mean of all genes in a sample after normalization. The Angiogenesis geneset included the following genes: JAG1, VIM, PDGFA, NRP1, ACTA2, TIMP1, JAG2, SNAI2, TGFBR3, VCAM1, NOTCH2, VEGFFC, ITGAV, FGFR, PDGFRB, VEGFA, BMP1, TGFB1, CD44, COL3A1. The inflammatory Response geneset included the following genes: MX1, IL4R, IL7, IRF9, LY6E, IL15, LAMP3, IRF2, CSF1, LGIS3BP, IFIT3, IFIT2, IRF7, CD74, CXCL9, CXCL10. IRF1, CD47. The immune suppression gene set included the following genes: FLT1, MRC1, TGFBR2, EPAS1, TGFB3, HAVCR2, TEK, VCAM1, ITGAM, CSF1R, CD4, CD163

Gene set variation analysis (GSVA) was also utilized due to both the size and heterogeneous nature of the datasets, as this technique works on a single sample basis as opposed to grouping samples into predefined classes to evaluate enrichment within a specific group (6). The GSVA implementation was used in the R/Bioconductor package GSVA v. 1.38.2. Twelve gene sets were selected via expert curation that adequately capture angiogenic, immunogenic and other key pathways.

Biomarker Assignment

Patient TME subtype classification was designed to inform potential future clinician-guided care regarding the selection of therapeutic modalities associated with post-treatment BOR status. For example, patients in the A subtype, with a high angiogenic, low immune active disease state, might respond to a therapy that targets or reduces angiogenesis (e.g. anti-VEGF), whereas a patient in the IA subtype, with low angiogenic but high immune activity, might respond to an immune checkpoint inhibitor (e.g. anti-PD-1). Alternatively, patient status as biomarker positive (“B+”, e.g. likely to respond) versus biomarker negative (“B-”, e.g. not likely to respond) could be assigned using combinations of TME subtypes, determined by the therapeutic mechanism of action (MOA). For example, IA + IS are both “high immune” and might be grouped to more appropriately include a greater number of patients for a given immune modulating therapy.

Supplementary Results

Supplementary Table 1. Datasets

Datasets for training, testing, performance evaluation and interpretation of the TME Panel model were defined at the outset. These included two real world datasets (RWD) from patient cohorts treated with either immune checkpoint inhibitors (Gastric-Immune) or anti-angiogenic targeted therapy (Gastric-Angio) for evaluating the predictive performance of TME subtype classification on approved drugs of known mechanism of action, and two clinical trial datasets to examine performance in additional disease indications, Mela-Immune and Ova-Angio.

Model stage	TME Panel Analysis	Cohort Name	Cohort Description	Data type	Patient Number	Tumor Type
Training	10x-cross validation	ACRG	Asian Cancer Research Group; comparable clinical history, no targeted therapy	Affymetrix array	N = 298	Gastric
Optimization	Feature transferability		Misc. biobank samples	RNA-seq	N = 1,099	Gastric, Colorectal, Ovarian
Panel & Algorithm Locked						
Testing	Response prediction	Gastric - Immune	RWD, 2-3L, ICI naive, immune checkpoint monotherapy	RNA-seq	N = 73	Gastric
		Gastric - Angio	RWD, heavily pre-treated 3L+, ramucirumab + chemo	RNA-seq	N = 49	Gastric
		Mela - Immune	Phase 1b clinical trial, ICI refractory, vidutolimod + pembrolizumab	RNA-seq	N = 38	Melanoma

		Ova - Angio	Phase 1b clinical trial, heavily pretreated, navicixizumab + paclitaxel	RNA-seq	N = 33	Ovarian
Explanation	Independent gene-set enrichment	Singapore	RWD, mixed lines of treatment	Affymetrix array	N = 192	Gastric
		TCGA - STAD	RWD, no targeted therapy	RNA-seq	N = 375	Gastric (Stomach adeno-carcinoma)
Exploration	Survival prognosis	CIT (Cartes d'Identite des Tumeurs)	RWD, mixed stage primary tumor samples	Affymetrix array	N = 566	Colorectal

Additional gene expression data sets were sourced for model validation, feature set (gene signature) optimization, as well as explanatory and exploratory analyses of the model (Table 1 and supplementary table 1). RNA-sequencing data were generated from FFPE slides of 1,099 patient samples—392 ovarian, 370 colorectal, and 337 gastric cancer—commercially sourced from a biobank repository (HTG Molecular Diagnostics, Inc). Microarray gene expression data from 192 gastric cancer samples of the "Singapore Patient Cohort" were downloaded from GEO (GSE15459)(7). Total-RNA-seq gene expression profiles from 375 stomach adenocarcinoma (STAD) specimens collected for The Cancer Genome Atlas (TCGA) were downloaded from the National Cancer Institute Genomic Data Commons Data Portal. Lastly, the gene expression profiles of 566 colorectal cancer patients from the Cartes d'Identite des Tumeurs (CIT) collection were downloaded from GEO (GSE39582)(8). Data sets were processed and analyzed by standard bioinformatics methods according to the data type and library chemistry (e.g. RNA-seq versus microarray), including appropriate transformations and normalizations as described in the Supplementary Materials. TCGA-STAD data enabled normalization evaluation and feature reduction to ensure robustness of assay type. The combined z score method (4), which considers z scores together for each signature set at each individual sample, was used to compute an activation index. Although there are several published measures for activation of pathway or signature in a given tissue (5), the combined z score method was selected because it is intuitive and may be platform independent. The z

score represents the difference in standard deviations between the error-weighted mean of the expression values of the genes in a pathway and the error-weighted mean of all genes in a sample after normalization.

Supplementary Table 2.

A summary table of demographic and clinical information referred to in the manuscript is provided as an Excel file.

Supplementary Figure 1. Data normalization and feature transferability

(A) All datasets were normalized to enable interoperability between the datasets. Transcripts per million, standardization (z-score) and per-gene quantile transformation were compared, with the latter demonstrating the most consistent results.

(B) A novel method for gene set reduction was employed to quantify the consistency of normalized expression values across various datasets for each gene in a feature set. Dubbed Feature Transferability, this method also enabled assessment of the extent to which each gene contributed to the separation of target variables (e.g. subtypes). As an illustrative example, Gene 1 in the schematic is generally consistent in its expression across all datasets and in its separation of TME phenotypes, while Gene 2 was not. Gene 2 would be removed from the feature set prior to model training.

1A

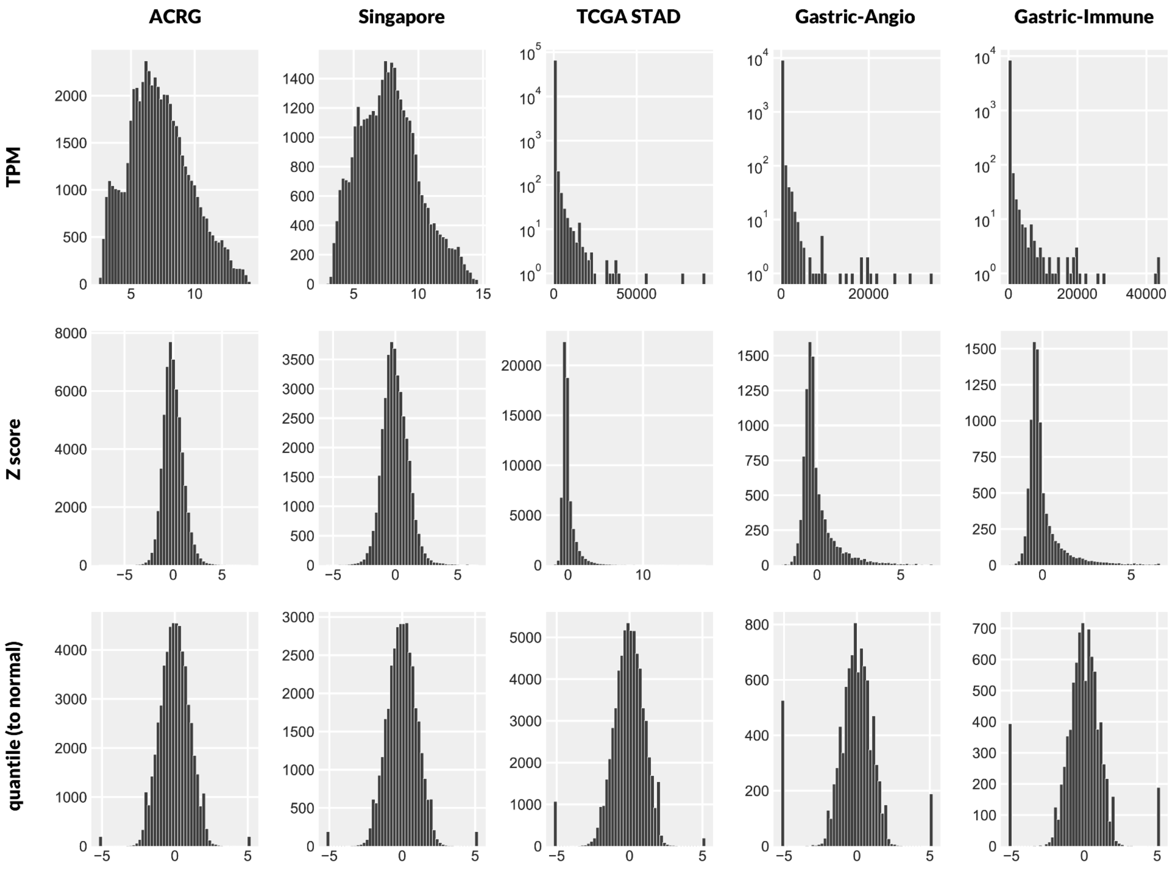
ACRG
Singapore
TCGA STAD
Gastric-Angio
Gastric-Immune
TPM
Z score
quantile (to normal)

1B

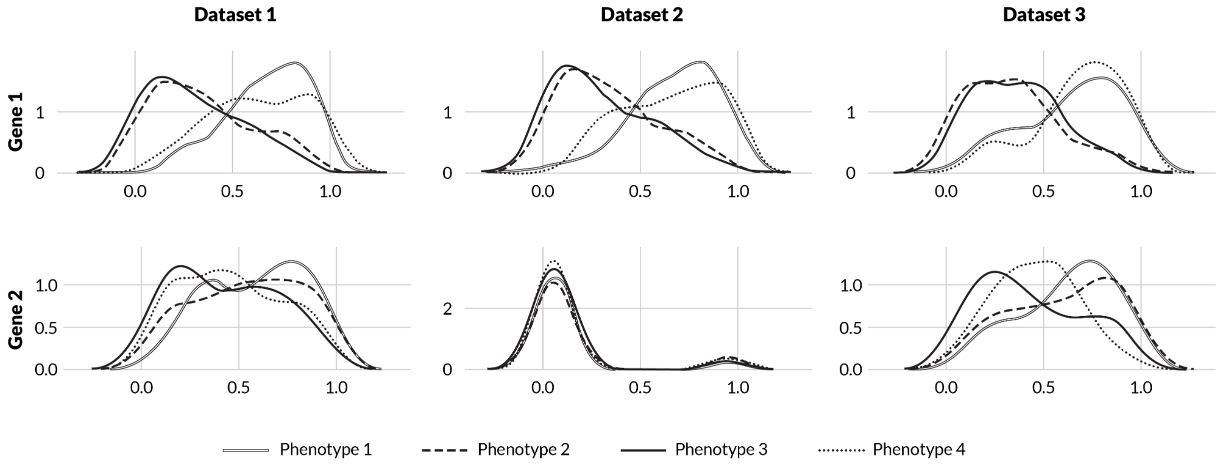
Dataset 1
Dataset 2
Dataset 3
Gene 1
Gene 2
Phenotype 1
Phenotype 2
Phenotype 3
Phenotype 4

Supplementary Figure 2. GSVA Enrichment Scores and Drug Response

GSVA enrichment scores were plotted for each patient sample and grouped according to their best overall response to drug. Data is shown for the most relevant hallmark signatures to each cohort (i.e. angiogenesis-related signatures for cohorts treated with anti-angiogenic agents). Box-whisker plots show each sample as an open circle, with the “x” representing the mean, and the horizontal line representing the median of each group.
(A-D) GSVA enrichment scores for non-responders and responders in the Gastric-Angio cohort, Ova-Angio cohort, Gastric-Immune cohort, Mela-Immune cohort respectively. Performance of the GSVA enrichment scores was also computed, samples were separated into biomarker positive (GSVA enrichment score > 0) and negative (GSVA enrichment score <0).

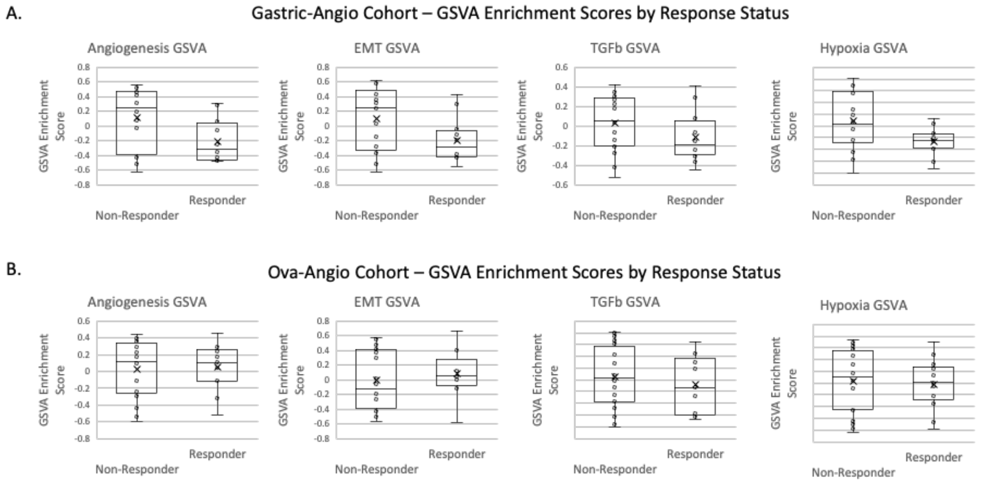

Gastric Angio	GSVA Geneset	ACC	AUC ROC	F1	Sensitivity	Specificity	PPV	NPV
	Angiogenesis	0.56 (27/48)	0.57	0.53	0.63 (12/19)	0.52 (15/29)	0.46 (12/26)	0.68 (15/22)
	Epithelial Mesenchymal Transition	0.58 (28/48)	0.58	0.52	0.58 (11/19)	0.59 (17/29)	0.48 (11/23)	0.68 (17/25)
	TGF Beta Signalling	0.60 (29/48)	0.61	0.56	0.63 (12/19)	0.59 (17/29)	0.50 (12/24)	0.71 (17/24)
	Hypoxia	0.40 (19/48)	0.41	0.38	0.47 (9/19)	0.34 (10/29)	0.32 (9/28)	0.50 (10/20)
Ova-Angio	Angiogenesis	0.50 (16/32)	0.52	0.5	0.62 (8/13)	0.42 (8/19)	0.42 (8/19)	0.62 (8/13)
	Epithelial Mesenchymal Transition	0.59 (19/32)	0.61	0.58	0.69 (9/13)	0.53 (10/19)	0.50 (9/18)	0.71 (10/14)
	TGF Beta Signalling	0.47 (15/32)	0.47	0.41	0.46 (6/13)	0.47 (9/19)	0.38 (6/16)	0.56 (9/16)
	Hypoxia	0.56 (18/32)	0.55	0.46	0.46 (6/13)	0.63 (12/19)	0.46 (6/13)	0.63 (12/19)

C.

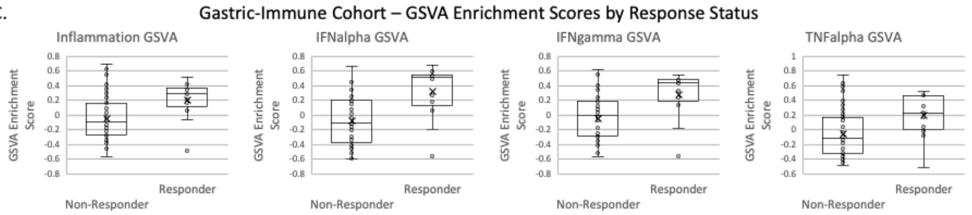

D.

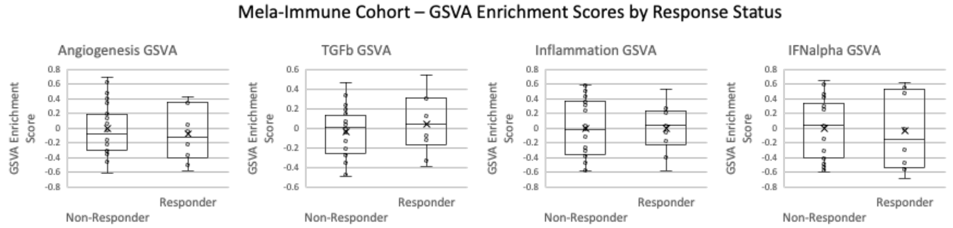

Gastric Immune	ACC	AUC ROC	F1	Sensitivity	Specificity	PPV	NPV
Inflammatory Response	0.66 (48/73)	0.73	0.47	0.85 (11/13)	0.62 (37/60)	0.32 (11/34)	0.95 (37/39)
Interferon Alpha Response	0.66 (48/73)	0.73	0.47	0.85 (11/13)	0.62 (37/60)	0.32 (11/34)	0.95 (37/39)
Interferon Gamma response	0.56 (41/73)	0.67	0.41	0.85 (11/13)	0.50 (30/60)	0.27 (11/41)	0.94 (30/32)
TNFA Signalling via NKFB	0.33 (24/73)	0.29	0.11	0.23 (3/13)	0.35 (21/60)	0.07 (3/42)	0.68 (21/31)

Mela-Immune	ACC	AUC ROC	F1	Sensitivity	Specificity	PPV	NPV
Angiogenesis	0.50 (19/38)	0.47	0.3	0.40 (4/10)	0.54 (15/28)	0.24 (4/17)	0.71 (15/21)
TGF Beta Signalling	0.50 (19/38)	0.5	0.34	0.50 (5/10)	0.50 (14/28)	0.26 (5/19)	0.74 (14/19)
Inflammatory Response	0.55 (21/38)	0.57	0.41	0.60 (6/10)	0.54 (15/28)	0.32 (6/19)	0.79 (15/19)
Interferon Alpha Response	0.55 (21/38)	0.57	0.41	0.60 (6/10)	0.54 (15/28)	0.32 (6/19)	0.79 (15/19)

Supplementary Figure 3. Prognostic Capabilities

The prognostic capabilities of the model were evaluated using recurrence free survival (RFS) and overall survival (OS). To visualize the relationship between TME subtype and survival Kaplan-Meier plots were generated. RFS was calculated for eligible cases as the length of time after primary treatment that the patient survived without any signs or symptoms of cancer. OS was similarly calculated for all eligible cases and was defined as the time from treatment initiation to date of death or last contact.

(A) Recurrence free survival of CRC patients from the historical CIT dataset analyzed by the Kaplan Meier method. Patients were stratified by TME subtype, which are colored according to the legend. n/N refers to the number of samples that have a recurrence event out of the total number of TME subtype allocations.

(B) Overall survival of late stage (stage 3-4 CRC patients from the historical CIT dataset analyzed by the Kaplan Meier method. Patients were stratified by TME subtype, which are colored according to the legend. The A subtype has the lowest median overall survival at 69 months

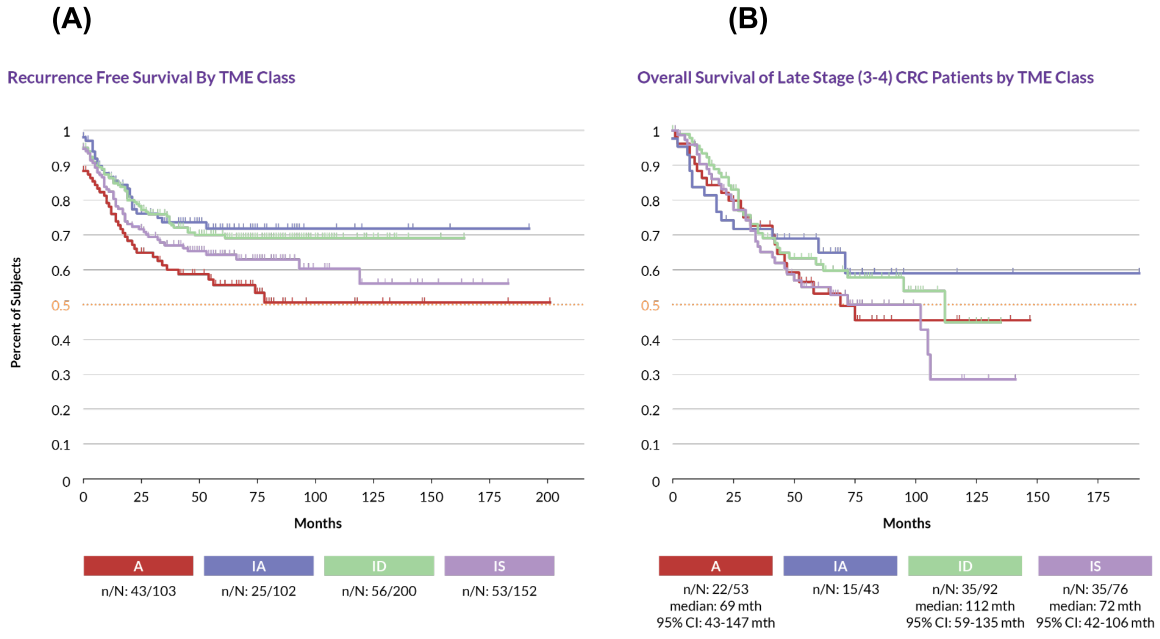

Supplementary References

1. Uhlik MT, Liu J, Falcon BL, Iyer S, Stewart J, Celikkaya H, et al. Stromal-Based Signatures for the Classification of Gastric Cancer. Cancer Res. 2016;76:2573–86.
2. Strand-Tibbitts K, Kim K, Hong JY, Kim ST, Lee J, Benjamin L. Working Towards Precision Medicine for the Tumor Microenvironment. SITC 2019 Poster. 2019;1.
3. Strand-Tibbitts K, Culm-Merdek K, Ausec L, Zganec M, Stajdohar M, Lee J, et al. Development of an RNA-based Diagnostic Platform Based on the Tumor Microenvironment Dominant Biology. SITC 2020 Poster. 2020;1.
4. Lee E, Chuang H-Y, Kim J-W, Ideker T, Lee D. Inferring Pathway Activity toward Precise Disease Classification. Tucker-Kellogg G, editor. PLoS Comput Biol. 2008;4:e1000217.
5. Levine BL, Humeau LM, Boyer J, MacGregor R-R, Rebello T, Lu X, et al. Gene transfer in humans using a conditionally replicating lentiviral vector. Proc Natl Acad Sci. 2006;103:17372–7.
6. Hänzelmann S, Castelo R, Guinney J. GSVA: gene set variation analysis for microarray and RNA-Seq data. BMC Bioinformatics. 2013;14:7.
7. Lei Z, Tan IB, Das K, Deng N, Zouridis H, Pattison S, et al. Identification of molecular subtypes of gastric cancer with different responses to PI3-kinase inhibitors and 5-fluorouracil. Gastroenterology. 2013;145:554–65.
8. Marisa L, de Reyniès A, Duval A, Selves J, Gaub MP, Vescovo L, et al. Gene expression classification of colon cancer into molecular subtypes: characterization, validation, and prognostic value. PLoS Med. 2013;10:e1001453.

Acknowledgements

The results in this manuscript are in part based upon data generated by the TCGA Research Network: https://www.cancer.gov/tcga.
